# Pediatric vascular anomalies with airway compromise

**DOI:** 10.1111/jop.13297

**Published:** 2022-04-06

**Authors:** François Gorostidi, Nicolas Glasson, Victoria Salati, Kishore Sandu

**Affiliations:** ^1^ Department of Otolaryngology and Head Neck Surgery Lausanne University Hospital (CHUV) Lausanne Switzerland

**Keywords:** airway management, lymphatic malformation, pediatric airway, subglottic hemangioma, tracheotomy, vascular anomaly, vascular malformation, vascular tumor

## Abstract

Vascular anomalies are rare lesions of diverse nature that may affect the head and neck region. Any mass in or around the upper airway has the potential to obstruct or compromise it. The absolute priority, before etiologic treatment, is the evaluation of the risk for the airway and its management. Prenatal diagnosis of an upper airway obstruction requires a planned delivery in a center having a specialized team experienced in managing a compromised feto‐neonatal airway, and who could perform an ex‐utero intrapartum treatment to secure the airway. Even after birth, the airway remains central in the patient's overall management. Signs and symptoms of airway compromise must be evaluated keeping in mind the specific requirements of infants and small children and being aware that rapid worsening may occur. The treatment is then tailored to the patient and his lesion with the goal of improving symptoms while avoiding treatment‐related complications. Maintaining reasonable expectations by the patient and families are part of a successful management. Cure is achievable for small and localized lesions, but symptom relief and mitigation of functional, esthetic and psychological impairments is the goal for large and complex lesions. If a tracheotomy was required, decannulation is one of the primary management goals.

## 1 INTRODUCTION

The term vascular anomaly describes a wide spectrum of lesions composed of vascular tumors and vascular malformations.^1^, ^2^ The widely accepted classification by the international society for the study of vascular anomalies (ISSVA)^1^, ^3^ excludes terms like “lymphangioma”, “cystic hygroma” and “arterio‐venous hemangioma”. The suffix “angioma” is now strictly restricted to vascular tumors histologically displaying increased cell proliferation. This classification eventually solved the confusion around this spectrum of pathologies.^3^ Both vascular tumors and vascular malformations can affect the head and neck region with potential functional and aesthetic impairments. Symptoms depend on the nature, size and the localization of the lesions and include airway compromise, obstructive sleep apnea, dysphagia, sialorrhea, impaired mastication, abnormal speech intelligibility, and facial deformities.^4^ When the buccal cavity, pharynx, larynx, trachea and mediastinum are affected, management of airway compromise is the priority before definitive treatment of the vascular anomaly itself. The airway must be secured if it is obstructed and protected from potential future obstruction to prevent any emergent situation. Indeed, symptoms can rapidly deteriorate with vascular anomaly growth or swelling in case of infection, trauma, intralesional bleeding and hormonal changes^5^ or as a result of the treatment itself.^4^ Ranging from unnoticeable to massive, perinatal and pediatric vascular anomalies with airway compromise represent a clinical challenge and requires timely management in specialized centers having multidisciplinary teams with experience in managing a difficult pediatric airway. The primary goal of the management is obtaining a “safe” airway that would then permit a case‐specific treatment. Beyond the life‐threatening airway obstruction, management may also deal with impaired oral feeding, orthognathic considerations, speaking skills and decannulation programs. Therefore, depending on the type of lesion and its extent, treatment goals may vary from complete remission to improvement of function and quality of life, while minimizing treatment‐related complications.

## 2 BASIC PRINCIPLES OF AIRWAY MANAGEMENT IN PEDIATRIC HEAD AND NECK VASCULAR ANOMALY

Extrinsic compression or intrinsic obstruction at any level of the airway can result in a compromised or critical airway. It is crucial to immediately appreciate the severity of respiratory distress to determine the degree of emergency: 1. *Mild–moderate* (increased work of breathing [WOB], child responsive to external stimulations, tachypnea, noisy breathing, mild to moderate chest retractions), 2. *Severe* (laborious WOB, cyanosis, nasal alar flaring, child non‐responsive to external stimulation, noisy breathing, severe neck‐chest‐abdominal retractions), 3. *Terminal* (exhausted child and reduced WOB, decreasing respiratory noises, decreasing retractions).^6^ The quality of the noise provides clues to the location of the airway obstruction while its intensity correlates, to a certain extent, with the degree of obstruction.^6^ An obstruction at the level of the larynx or trachea usually produces a high‐pitched sound, called stridor, which can be inspiratory (supraglottic), biphasic (glottic/subglottic) or expiratory (trachea). An obstruction at the level of the pharynx or the oral cavity produces more of a low pitch noise, analog to snoring, usually inspiratory, and is called stertor.^6^ Depending on the location of the vascular anomaly, the degree of obstruction can worsen in a supine position, for example with lesions involving the base of the tongue. Feeding difficulty and failure to thrive is also an indicator of the severity of the airway obstruction.

The nature of the lesion (compressible or not), together with its localization (endoluminal or extraluminal) has important implications in the perinatal and post‐natal management of the compromised airway. As a general principle, in experienced centers, tracheotomy should be avoided or delayed as much as possible to prevent any further tracheal damage and to avoid tracheotomy‐related impairements.^6^, ^7^, ^8^ Noninvasive ventilation and/or tracheal intubation can temporarily secure a compromised airway while providing time to relieve an obstruction, for example with decompressive aspiration or early surgery. Complex and/or multilevel lesions obstructing the upper airway are difficult to treat and are best addressed under a tracheotomy‐secured airway. Performing a tracheotomy in case of a large vascular anomaly of the neck can itself be challenging. In any case, the tracheotomy should always be performed with the airway secured by either a tracheal intubation or rigid bronchoscopy. Surgical technique, multidisciplinary care, parents support and correct decannulation protocols are then crucial to minimize tracheotomy‐related complications and impairements.^6^, ^9^ Decannulation then becomes one of the primary goals of treatment.

## 3 AIRWAY MANAGEMENT IN VASCULAR ANOMALIES DIAGNOSED IN UTERO

Progresses made in prenatal screening with ultrasound (US) and magnetic resonance imaging (MRI) allow in utero detection of fetal head and neck masses with potential airway compromise.^10^, ^11^ Even if the diagnosis can be inaccurate,^12^ any suspicion should evoke a multidisciplinary evaluation to plan a safe perinatal airway management, sometimes with an ex‐utero intrapartum treatment (EXIT) procedure. An EXIT procedure provides up to 90 minutes to access the airway under utero‐placental circulation, but these require highly trained teams^13^ and may put the mother at risk of complications including severe uterine hemorrhage.^14^ Congenital high airway obstruction syndrome (CHAOS), which has specific signs^15^ such as polyhydramnios and inverted diaphragm, is the only true intrauterine upper airway obstruction. Fetal neck masses responsible for a CHAOS always require an EXIT procedure to secure the airway before any consideration of etiologic treatment. Lymphatic malformation (LM) is the most frequent neck mass diagnosed in utero, followed by teratoma.^16^ An EXIT procedure should be restricted to selected cases to avoid unnecessary maternal and fetal risks.^17^ Because most vascular anomalies are soft and/or compressible, CHAOS is unlikely, and most patients can be intubated through laryngoscopy or rigid bronchoscopy at birth. In a series of 112 fetuses with prenatal diagnosis of cervical LM, 13 (11%) were delivered through an EXIT procedure.^17^ The criteria to perform an EXIT procedure were deviation, compression or obstruction of the airway and involvement of the floor of mouth and tongue. Of those 13 fetuses, 11 could be intubated with laryngoscopy (7) or rigid bronchoscopy (4), 1 required a tracheotomy, and one with a massive involvement of the face and neck died after parental refusal of a tracheotomy.^17^ The authors conclude that EXIT procedure remains the gold standard, in specialized centers, to address prenatal LM with high suspicion of airway compromise.^17^

## 4 AIRWAY MANAGEMENT IN VASCULAR ANOMALIES DIAGNOSED AFTER BIRTH

### 4.1 Vascular tumors

The list of vascular tumors is large,^3^ and includes infantile hemangioma (IH), congenital hemangioma, kaposiform hemangioendothelioma and pyogenic granuloma. Because of space constraints, this review will only discuss IH, the most frequent vascular tumor impacting the airway.

#### 4.1.1 Infantile hemangioma (IH)

IH is the most frequent tumor of infancy affecting up to 5% of children,^18^ 60% of which in the head and neck region,^19^ with a 2–3:1 female predominance.^3^, ^6^ IH can impact the airway when located in the oral cavity, pharynx or larynx. The subglottic localization of IH, termed subglottic hemangioma (SGH), represents 1.5% of congenital anomalies of the larynx^6^, ^20^ and is classically linked to segmental cutaneous IH, especially with a bilateral beard distribution (association 10‐30%).^21^, ^22^, ^23^ Although only 1‐2% of patients with cutaneous IH have a silent SGH, it is prudent to look for it before patients present with a critical airway. 30% of beard distributed IH have a PHACE syndrome (**P**osterior fossa anomalies, **H**emangioma, **A**rterial lesions, **C**ardiac abnormalities/aortic coarctation, and **E**ye abnormalities), these patients should be thoroughly investigated.^24^ Early diagnosis should prevent high rate of repeated hospitalizations (30%) and mortality (2%) described before the use of Propranolol.^23^, ^25^ However, up to 55% of subglottic hemangiomas present as recurrent/persistent/severe croup without skin lesions,^26^ often leading to a delayed diagnosis. The typical clinical course of IH is a proliferative phase during the first months of life reaching its maximal size around 9–12 months, followed by a spontaneous involution by 24–30 months.^27^, ^28^ The mean age of diagnosis of an airway IH is around 3.6 months.^29^ A subglottic hemangioma can be suspected on awake fiberoptic laryngoscopy and confirmed with angio‐CT as an intensely contrast‐enhancing, lobular, well delimited mass.^21^ MRI is also diagnostic with the advantage of avoiding infant irradiation but it often requires a sedation.^21^ With the disadvantages of a general anesthesia for subglottic hemangioma that are now essentially treated medically, direct laryngoscopy is usually diagnostic, and it provides the opportunity to precisely assess the risk for a critical airway. On rod lens endoscopy, subglottic hemangioma presents as smooth submucosal, compressible, pink or blue mass with surface telangiectasias. Biopsy is often not necessary but positive GLUT1 staining confirms the diagnosis.^3^

A subglottic hemangioma is a time bound self‐limiting vascular tumor that involutes by the 3rd birthday of the child. Therefore, the treatment should be directed towards securing and maintaining a safe airway and avoiding sequelae of the treatment. Historically, treatment options were: tracheotomy followed by *wait‐&‐watch* policy until spontaneous involution occurs followed by decannulation^30^; laser (CO_2_ ^31^ or KTP^32^) ablation, endoscopic microdebrider,^33^ intra‐lesional and systemic steroids and open neck surgery.^34^, ^35^ Open surgical resection offers a high degree of success, with more than 90% patients remaining symptom‐free from their subglottic lesion, not requiring a tracheotomy when primarily employed as a single‐staged technique.^35^

**FIGURE 1:**
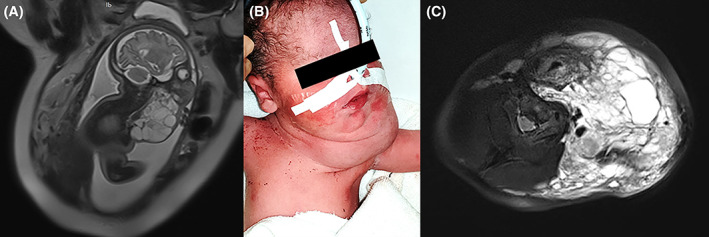
(A) Fetal MRI showing a large lymphatic malformation with high suspicion of airway compromise (deviation of the airway, floor of mouth and base of tongue involvement). (B) newborn intubated after the EXIT procedure. (C) axial MRI view of the lymphatic malformation at 3 months of age with deviation of the airway to the right. Despite massive cervical involvement, the case was managed without the need for a tracheotomy

Since the initial report in 2008,^36^ the use of propranolol has dramatically modified the management of IHs including subglottic hemangioma.^37^, ^38^ Propranolol is a cardio‐selective beta blocker affecting the subglottic hemangioma growth in 3 ways: (1) vasoconstriction, (2) apoptosis or programmed cell‐death and (3) reduction of the vascular endothelial growth factors. After a pretreatment assessment protocol,^39^ Propranolol is started at a dose of 2 mg/kg/day in 3 divided doses. Maintenance dose of 1 mg/kg/d is continued for 6–12 months, with no definite agreement among experts regarding the treatment duration. Propranolol‐resistant subglottic hemangioma^27^ (as is observed in PHACE syndrome or beard IH) can be treated by Acebutol^40^ or surgery and refractory cases after surgery can be treated with propranolol.^39^ Despite the paradigm change in the treatment of subglottic hemangioma, the authors believe that primary resection by open surgery or CO_2_ laser ablation still has a role for the primary treatment of properly selected lesions as results can be excellent while avoiding long term medical treatments with potential side effects (Figure 1).

### 4.2 Vascular malformations

**FIGURE 2:**
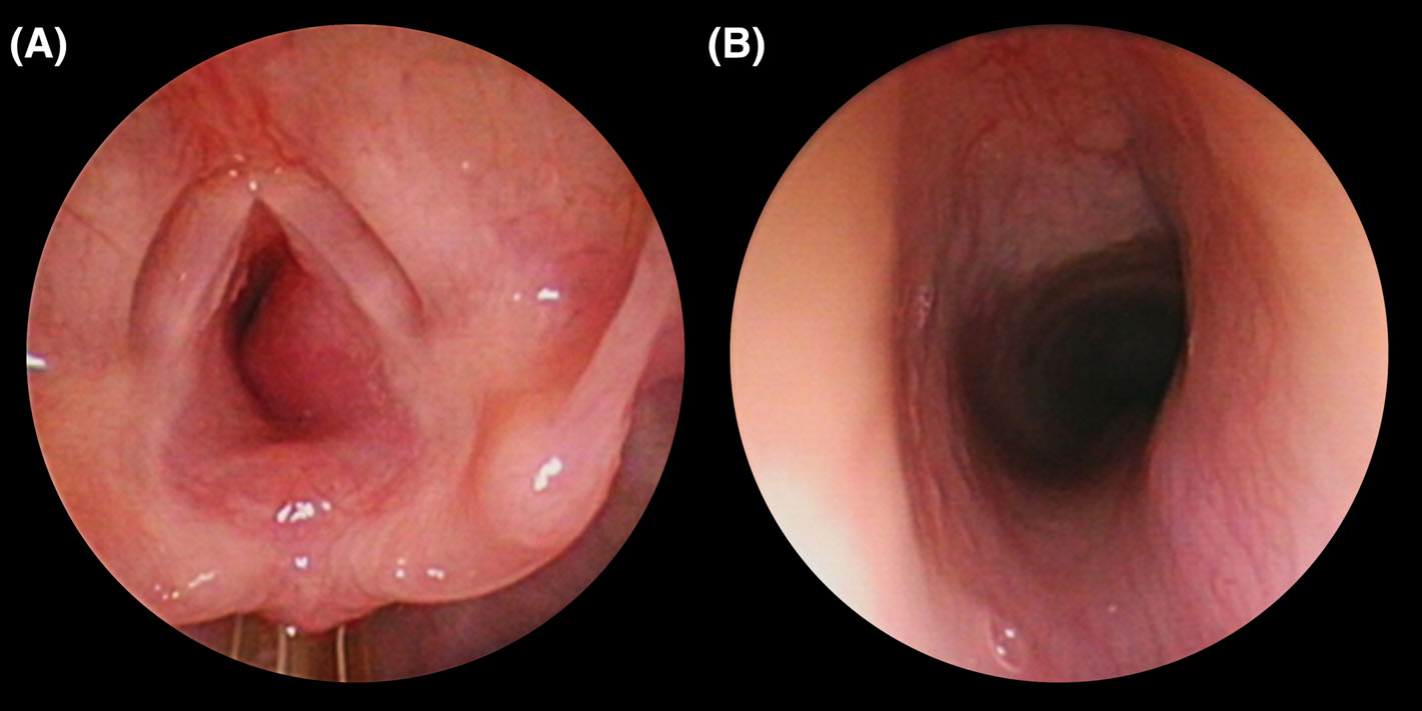
Three‐months‐old child which presented with a biphasic stridor, and signs of respiratory distress without cyanosis. (A) 0° rod lens telescope view showing a smooth submucosal subglottic mass with 90% obstruction of the lumen suggestive of subglottic hemangioma. Propanolol was started immediately, and non‐invasive ventilation could be weaned off after 48 h. (B) 0° rod lens telescope view showing almost complete response after 6 months of treatment. A remnant of the hemangioma obstructing only 10% of the lumen is still visible

Vascular malformations are vascular lesions made of dysplastic vessels lined with normal endothelium which are always present at birth and grow proportionally to the patients with exacerbations/remissions. They are classified as simple or mixed depending on the cells composing it and as low‐flow or high‐flow.^1^ Each type of vascular malformation represents a distinct therapeutic challenge. The pathophysiology underlying the development of vascular malformations is beyond the scope of this review but recent insights in the biology of vascular malformations have changed the diagnostic accuracy and have opened the path for targeted medical therapies.^41^ Because of space restrictions, only the simple types of vascular malformations will be discussed here (Figure 2).

#### 4.2.1 Lymphatic malformations

With a reported incidence of 1.2 to 2.8 per 1000^42^ live‐births, LMs are the most frequent lesion with potential airway compromise diagnosed on prenatal screening.^43^ Always present at birth, although not always obvious, LMs grow proportionally to the patient with episodes of swelling/remission linked to infection, and trauma.^44^ Only around 60% of LMs are evident at birth and 80%–90% are diagnosed by the ae of two.^45^ They exist in any body location but the head and neck is most frequently affected, with up to 73% of upper aerodigestive tract involvement.^44^ In a retrospective series of 141 patients with upper aerodigestive tract LM, the buccal cavity was affected in 75%, the oropharynx in 36%, the hypopharynx in 6%, the parapharynx in 30%, and the retropharnyx in 9%.^44^ The supraglottis was only involved in case of large LM involving other subsites, most frequently the buccal cavity and/or pharynx. There were no involvement of the glottis and subglottis.^44^ All these localizations can result in airway obstruction, especially when the lesion is in direct contact with the airway.^46^ Airway compromise from mediastinal LM is also described.^47^

Head and neck LMs are staged according to De Serres^45^ as stage I, unilateral infrahyoid; stage II, unilateral suprahyoid; stage III, unilateral infrahyoid and suprahyoid; stage IV, bilateral suprahyoid; stage V, bilateral infra and suprahyoid.^45^ The risk of complications and recurrences/residual disease after treatment increases with higher stages.^41^ LMs are further classified radiologically as microcystic (<2 cm cysts), macrocystic (>2 cm cysts) and mixed. Stage I‐III, all unilateral, account for more than 85% for cervical LM, they cause minimal functional impairments, have better outcomes, and show spontaneous regression in up to 30% of cases.^45^, ^48^ Stages IV and V account for 15% of cervical LM, tend to be microcystic and often create functional impairments including airway compromise.^45^, ^48^ A distinct classification exists for microcystic LM of the tongue with poorer prognosis in higher stage lesions.^48^, ^49^ Macrocytic lesions tend to displace adjacent structures with potential extrinsic airway obstruction. Microcysts are known to be more invasive to surrounding structure, harder to treat and more prone to recurrences/persistent disease.^48^

**FIGURE 3:**
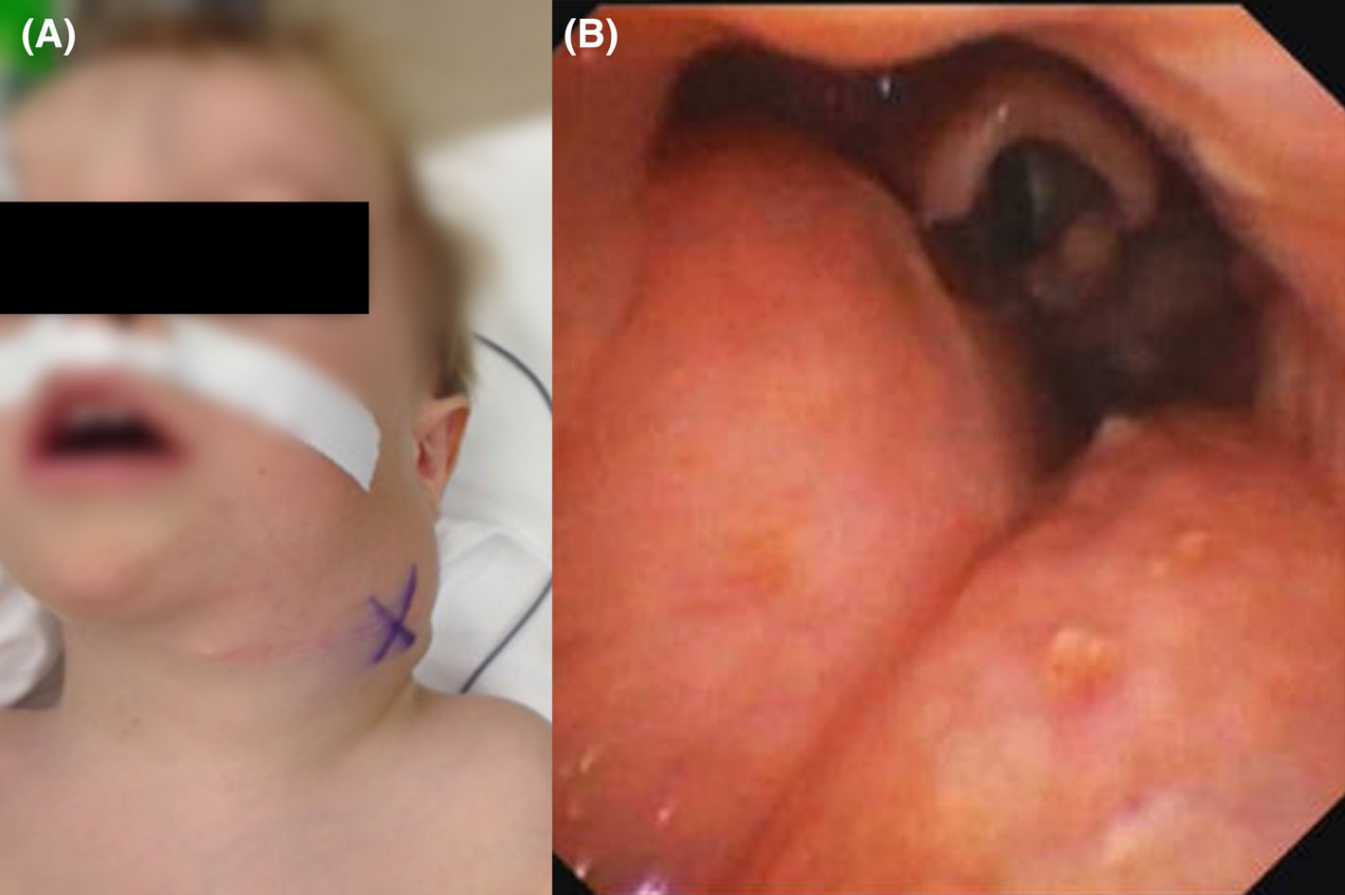
Two‐years‐old child with a left cervical macro‐microcystic lymphatic malformation with mediastinal extension. The child suffered of severe obstructive sleep apnea syndrome necessitating non‐invasive ventilation and had a neck exploration prior to his referral to our clinic. (A) external view, (B) 0° rod telescope view showing extrinsic pharyngeal obstruction by the lymphatic malformation

After birth, the diagnosis is suspected on clinical exam and confirmed with US or/and MRI. Biopsy is often not necessary but can confirm the diagnosis.^48^ LMs usually present as skin/mucosal yellow‐blue soft mass with episodes of swelling and redness in case of inflammation. Tongue LMs present with bluish vesicles of the tongue surface with or without magroglossia.^49^ Deep LMs present with non‐tender, soft mass with symptoms related to the size and localization (Figure 3).

**FIGURE 4:**
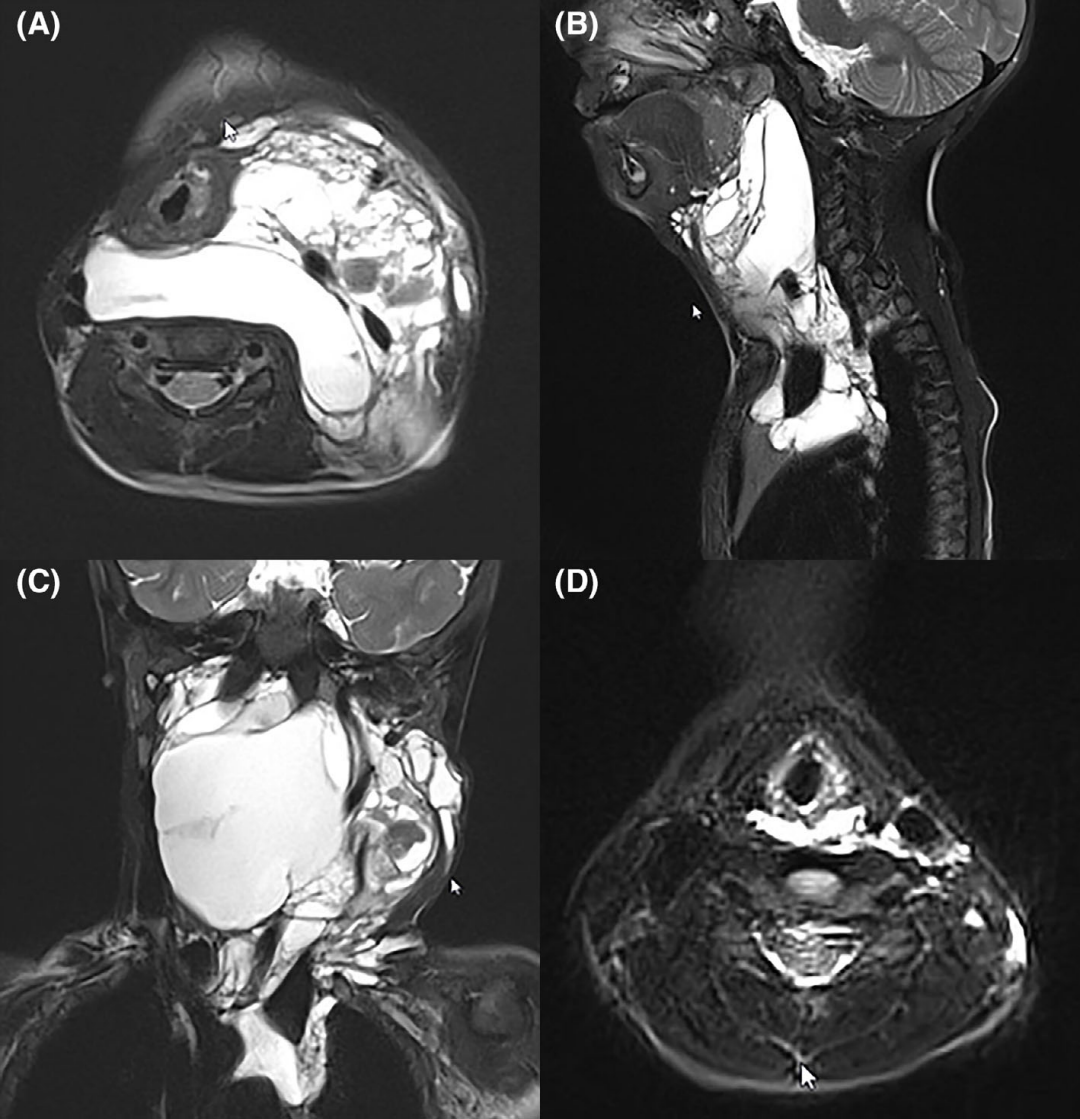
Same child as in Figure 3. A (axial), B (sagittal) and C (coronal) preoperative MRI at 2 years of age. The mixed lymphatic malformation insinuates between cervical plans and causing moderate–severe degree of extrinsic airway obstruction and mediastinal extension. (D) Axial MRI at 7 years of age, following surgery and intralesional sclerotherapy with Bleomycin

In a retrospective survey series of 518 patients with head and neck LM, 14% (73/518) presented symptoms of airway obstruction and 8.3% (43/518) required a tracheotomy ‐ 74% (32/43) of which during the first year of life.^46^ Decannulation seemed less likely if the tracheotomy was necessary during the first year of life and the global decannulation rate was 40%.^46^ Airway obstruction by infection/swelling, known to be temporary, are best managed by noninvasive ventilation or temporary tracheal intubation to avoid unnecessary tracheotomies and their consequences.^8^ However, with large LM with significant macroglossia, glossoptosis, diffuse subglottic involvement, wide pharyngeal involvement, it is safer to secure the airway with a tracheotomy, making it known to the parents that the goal of decannulation cannot always be achieved (Figure 4).

The type and extent of the disease are prognostic factors with regards to achieving optimal airway patency and influence the treatment choices.^50^ Results with both sclerotherapy and surgery are better in isolated lesions that are macrocystic, of any size, and worst when lesions are microcystic, involving the parotid gland, laryngopharynx, oral cavity and tongue. In most cases a staged multimodal strategy, including several rounds of surgery and sclerotherapy, is chosen because most lesions are mixed.^51^ Ideally, complete surgical excision should be performed; however, subtotal excision may be more appropriate for patients in whom complete excision has an unacceptably high risk of long‐term neurovascular and functional morbidity. The majority of laryngopharyngeal and tongue LM are microcystic and will necessitate surgery. Radical surgery in these two sites should not be done to avoid long‐term debilitating sequelae, meaning that multiple procedures may be required to maintain a safe airway and attempt decannulation. Surgery after primary sclerotherapy will be difficult and fraught with intra‐ and post‐operative complications due to extreme cicatrisation.^52^ Lesions that are partially excised can be secondarily treated with sclerotherapy with various agents.^52^ Transmucosal bleomycin sclerotherapy can treat residual disease within the airway in conjunction with CO_2_ laser resection under direct laryngoscopy.^53^ Bleomycin has low scarring and edema complications but inappropriate CO_2_ laser use can lead to airway stenosis.^53^ In general, sclerotherapy will induce inflammation and oedema and hence should be avoided or used with extreme care in lesions that are in close contact with the airway.

**FIGURE 5:**
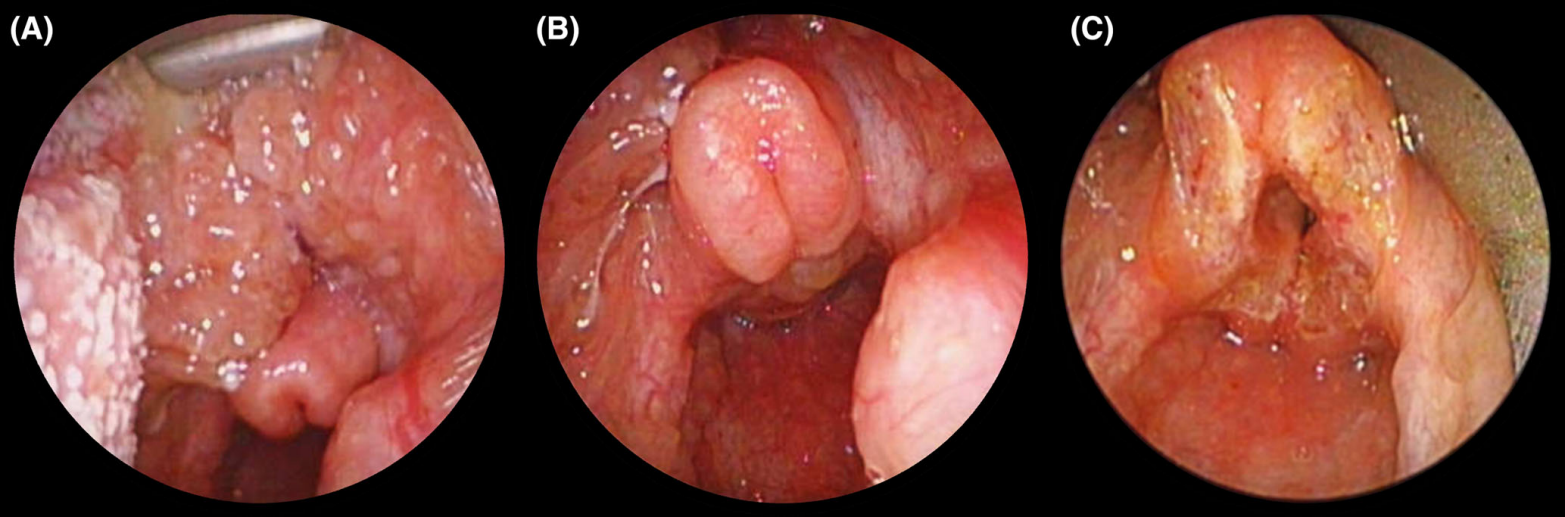
Two years old child with a pharyngolaryngeal macro‐microcystic lymphatic malformation (LM) necessitating a tracheotomy at 3 weeks of life. (A and B) endoscopic status after 6 sessions of sclerotherapy with ethanol leading in a 70% decrease of size of the lesion. A large lesion of the base of the tongue extending to the supraglottis, especially the right side of the epiglottis and right aryepiglottic fold leading to severe airway obstruction. (C) result after CO_2_ laser supraglottoplasty. After several more endoscopic treatment including base of the tongue resection and epiglottopexy, the child was eventually decannulated

The choice of primary treatment between surgery and sclerotherapy remains controversial and should be discussed on a case to case basis.^41^ Systemic antibiotics and corticosteroids are used to treat lymphangitis‐related swelling and high dose corticoids to prevent fibrosis after surgery and sclerotherapy. Sirolimus, an m‐TOR (mechanistic‐Target Of Rapamycin) inhibitor, may be effective in the cases of extensive lymphatic malformations which may require several rounds of sclerotherapy, but long term outcomes are yet to be described (Figure 5).^41^, ^54^

#### 4.2.2 Venous malformations

Venous malformations (VMs) are the third most common VA of the head and neck.^27^ They are bluish, soft, compressible lesions that increase with Valsalva and supine position. VMs can affect all subsites of the upper aero‐digestive tract from lips to subglottis with symptoms related to their size and localization (facial deformity, malocclusion, dental misalignment, impairment of mastication, speech and swallowing). They can be uni‐, multifocal or diffuse and grow proportionally to the patient with episodes of exacerbation linked to infection, trauma, hormonal changes, or intralesional thrombosis.^5^ Airway compromise may result from direct obstruction or bleeding. VMs of the face and buccal cavity are obvious but pharyngeal and laryngeal lesions may be present later with pain, dysphagia, dysphonia, and stridor worsening with Valsalva or in supine position. 70% of patients with facial cutaneous VMs have another lesion in the airway,^4^ hence awake fiberoptic pharyngo‐laryngoscopy should be done in every such patient. Superficial lesions can be diagnosed on history and clinical examination. Deep and extensive lesions require MRI, and sometimes ultrasound to properly assess the relations to surrounding structures.^5^ Most superficial VMs do not require treatment in the first years of life, unless they have functional repercussions such as in the orbit, facial musculoskeletal framework or in/around the airway.

The need to secure the airway with a tracheotomy depends on the localization and the extent of the disease, especially within the upper airway. The presence or absence of tracheotomy also influences the treatment strategy. Limited mucosal disease can be addressed without tracheotomy by fiber laser (Nd:YAG/KTP/Diode) spotting to induce fibrosis in the lesion. Direct sclerotherapy with bleomycin, causing minimal swelling, is also an efficient option in such cases.^55^

Large/diffuse mucosal lesions are better addressed with the airway protected by a tracheotomy. In these cases the treatment is multimodal (medical treatment, surgery, sclerotherapy, targeted therapy) with the goal of relieving symptoms while minimizing treatment‐induced complications.^56^ Multiple sclerosing agents can be used and sclerotherapy has shown good efficacy but with some complications.^57^ Much care must be taken when planning sclerotherapy in a VM close to the airway as these lesions are known to swell more than LMs after sclerotherapy. Surgery is a good alternative for small well defined lesions (through excision) and large VMs in close proximity to a critical structure (by debulking) and should be favored if crucial structures are involved or as part of the multimodal treatment.^41^, ^58^ Prior embolization may simplify surgical resection and debulking. Sirolimus, as a targeted therapy, can reduce the size and symptoms of VMs.^59^

#### 4.2.3 Capillary malformations

Capillary malformations (CMs) are red‐purple lesions, present at birth, made of ectatic vessels limited to the dermis/submucosal layer. Most are sporadic, but some are part of syndromes such as Sturge–Weber which is associated with facial CM.^5^ With time, they usually get thicker and induce hypertrophy of the surrounding soft tissues, in some cases resulting in severe disfigurement. They can be extensive and often affect the head and neck, especially in the lips, buccal cavity, and oropharynx^4^, ^5^ with functional impairments related to their localization and extent (airway obstruction, sleep apneas, facial deformity, impaired mastication, etc). Sleep apnea surgeries such as tonsillectomy and base of the tongue resection can help relieve some patients. The diagnosis is clinical as CM (superficial) sometimes remain unnoticeable on MRI and ultrasound,^5^ however, MRI remains paramount to characterize extensive disease. The mainstay of treatment is flashlamp‐pumped pulsed dye laser, which should be started as early as possible to maximize the results.^60^ In extensive CM, remaining soft tissue hypertrophy sometimes requires surgical resection or CO_2_ laser ablation.^4^, ^5^

## 5 CONCLUSIONS

Multiple vascular tumors and vascular malformations can affect the head and neck region with potential airway compromise. Early and accurate diagnosis allow the early initiation of a lesion‐specific treatment, minimizing morbidity and mortality. Airway management is the absolute priority before etiologic treatment. Fetuses with prenatal signs of upper airway obstruction should be evaluated in centers experienced in managing difficult feto‐neonatal airways and selected cases should be delivered through an EXIT procedure to secure the airway. After birth, unnecessary tracheotomies should be avoided; however, many lesions will require long and multimodal treatments with a tracheotomy securing the airway. In these cases, decannulation becomes one major goal of the treatment. The complex and various nature of vascular tumors and anomalies requires treatment selection to be tailored to the individual patient according to the disease characteristics and the functional consequences. Recurrence and incomplete treatment are common, but timely management by multidisciplinary team approach is key to the best possible outcomes.

## CONFLICT OF INTEREST

None.

### PEER REVIEW

The peer review history for this article is available at https://publons.com/publon/10.1111/jop.13297.

## Data Availability

The data is available on request from the authors (The data that support the findings of this study are available from the corresponding author upon reasonable request.)
